# Relationship Between Routine Preoperative Chest CT-Based Cardiac Parameters and Recanalization After Mechanical Thrombectomy in Patients with Acute Ischemic Stroke

**DOI:** 10.3390/jcm15124446

**Published:** 2026-06-09

**Authors:** Weizhi Xia, Yingbao Huang, Qi Chen, Xue Wang, Zhihan Yan, Wenru Zhang

**Affiliations:** 1 Department of Radiology, The Second Affiliated Hospital of Wenzhou Medical University, Wenzhou 325000, China; 2 Department of Radiology, The First Affiliated Hospital of Wenzhou Medical University, Wenzhou 325000, China; 3 Department of Radiology, Taizhou Hospital of Zhejiang Province Affiliated to Wenzhou Medical University, Taizhou 318000, China

**Keywords:** acute ischemic stroke, thrombectomy, recanalization, routine preoperative chest CT, cardiac parameters

## Abstract

**Purpose**: Acute ischemic stroke (AIS) is the most prevalent stroke subtype. Given the brain–heart interaction, this study investigated the association between cardiac parameters on admission routine preoperative chest CT and recanalization following thrombectomy in AIS patients. **Method**: We retrospectively analyzed 215 AIS patients (August 2018–June 2022) who underwent admission of none contrast chest computed tomography (NCCT) and thrombectomy within 24 h. Successful recanalization was defined as modified Treatment in Cerebral Ischemia (mTICI) score 2b-3. Multivariable logistic regression identified independent predictors. A nomogram was developed and validated using ROC, calibration, and decision curve analyses. **Result**: The cohort had a median age of 72 years; 63.7% were male. Hypertension (65.1%), atrial fibrillation (25.1%), and pleural effusion (56.3%) were prevalent. Successful recanalization occurred in 172 patients (80%). Independent predictors included mean arterial pressure (OR: 1.022, CI: 1.003–1.041, *p* = 0.025), left pulmonary artery diameter (OR: 0.838, CI: 0.733–0.958, *p* = 0.010), RV/A ratio (standardized) (OR:1.908, CI: 1.293–2.817, *p* = 0.001), septal angle (OR: 1.055, CI: 1.018–1.094, *p* = 0.004), and intraventricular septal angle (OR: 0.973, CI: 0.952–0.995, *p* = 0.015). The model achieved an AUC of 0.774 (*p* < 0.001) with strong calibration and net benefit. **Conclusions**: Cardiac parameters on routine preoperative chest CT correlate with recanalization following thrombectomy in AIS patients. The developed nomogram offers a reliable tool for clinical risk stratification.

## 1. Introduction

Acute ischemic stroke (AIS) represents the second leading cause of death and the third leading contributor to global disability [1]. Ischemic stroke is its predominant subtype [2]. Mechanical thrombectomy (MT) has emerged as a standard intervention for AIS patients with large vessel occlusions, promoting rapid cerebral reperfusion [3], and demonstrated superiority over intravenous thrombolysis alone [4,5,6]. Achieving recanalization, defined as modified Thrombolysis in Cerebral Ischemia (mTICI) scores of 2b-3, correlates significantly with favorable clinical outcomes [7]. Nonetheless, recanalization success rates exhibit considerable heterogeneity [8,9], prompting the need to identify reliable predictive factors to guide individualized therapy and optimize clinical decision-making.

Previous investigations have examined a range of variables affecting recanalization [10,11,12,13], including demographic attributes, comorbid conditions, laboratory indices, and advanced imaging features. Research has delved into clot characteristics, collateral flow, and treatment delays; however, many parameters entail specialized assessments that are not always feasible in acute scenarios [14].

Recent investigations have explored the brain–heart interaction after acute ischemic stroke [15,16]. Cardiovascular disease is regarded as the main predisposing risk factor for acute ischemic stroke (AIS) [17]. Moreover, AIS can contribute to impaired cerebral autoregulation, thus making cerebral blood flow directly dependent on cardiac function [18,19]. Cardiac dysfunction can both worsen the pre-existing cerebral damage and cause a new brain injury [20]. In the context of the brain–heart axis following acute ischemic stroke (AIS), cardiovascular dysfunction plays a pivotal role in determining patient prognosis, as the loss of cerebral autoregulation makes cerebral perfusion directly dependent on cardiac performance, thereby worsening cerebral outcomes. However, prior research has largely depended on CTA, cardiac MRI, echocardiography, SPECT, or PET/CT to evaluate cardiac function [21]. Due to the limitations of examination, these imaging assessment methods are not suitable for every ASI patient. In clinical practice, routine preoperative chest CT, routinely obtained during AIS patients upon admission, affords incidental cardiac and great vessel visualization [22], which may mirror cardiovascular status and hemodynamic dynamics, without incurring extra costs or radiation. Despite this potential, minimal research has exploited cardiac factors assessed through routine preoperative chest CT for predicting MT outcomes.

Therefore, this study aims to evaluate the utility of cardiac indicators derived from routine preoperative chest CT as a reliable parameter for assessing recanalization in AIS patients undergoing MT.

## 2. Method

### 2.1. Patients

**Figure 1:**
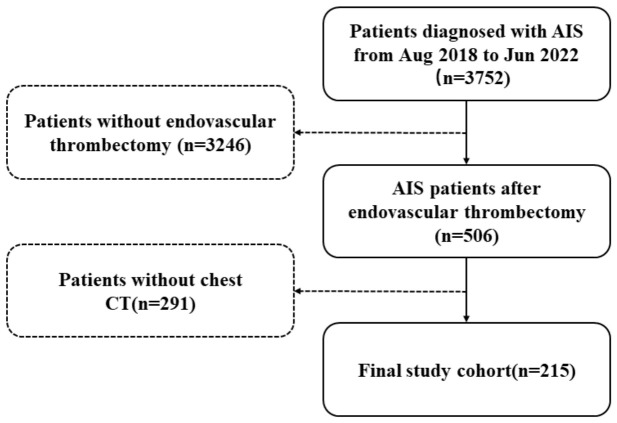
Flow diagram of exclusions and final study cohort.

This study employed a retrospective approach, and participants were recruited from a tertiary hospital between August 2018 and June 2022. Inclusion criteria were as follows: (1) Patients mandated a diagnosis of acute ischemic stroke (AIS), (2) the availability of comprehensive imaging and clinical data recorded in the electronic medical records, (3) completion of a non-contrast chest CT scan after hospital admission but prior to mechanical thrombectomy, (4) image coverage from the thoracic inlet to the diaphragm and slice thickness suitable for measurement (typically ≤5 mm). Exclusion criteria were as follows: (1) age below 18 years, (2) absence of mechanical thrombectomy within 24 h of symptom onset, (3) lack of none contrast chest computed tomography (NCCT) scans or missing images post-admission, and (4) confirmation of cerebral hemorrhage through brain CT after admission (Figure 1).

### 2.2. Clinical Variables and Outcome Indicators

Demographic variables encompassed gender and age. Clinical variables included hypertension, diabetes mellitus, history of smoking and alcohol consumption, cardiac surgery, or previous stroke, as well as the use of antiplatelet or anticoagulation medications, among others. Coexisting medical conditions comprised atrial fibrillation and coronary artery disease, among others. Laboratory indicators encompassed routine blood count, coagulation function, mean arterial pressure, temperature, heart rate, admission NIHSS (National Institutes of Health Stroke Scale) score [23], admission GCS (Glasgow Coma Scale) score [24], and abnormal electrocardiogram findings, among others. All clinical information was extracted from the electronic medical records. The outcome indicators were defined based on patient recanalization status, where in successful recanalization (SR) post-thrombectomy was characterized by an mTICI grade of 2b-3, while unsuccessful recanalization (USR) was defined by an mTICI grade of 0-2a. These determinations were made by clinicians during the surgical procedure and documented in the initial postoperative records. Based on the recorded results, we categorized the participants into the USR group and the SR group.

### 2.3. Cardiac Imaging Variables

**Figure 2:**
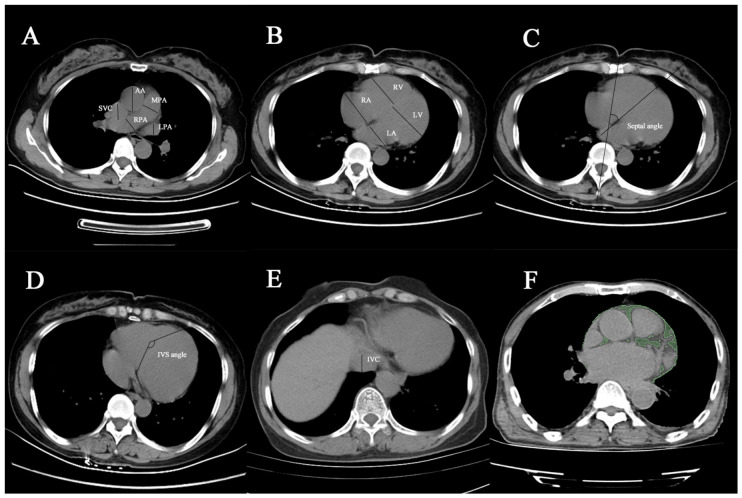
Diagram showing the methodology of calculation of the cardiac NCCT parameter. (**A**): Measurement of AA, MPA, RPA, LPA and SVC. (**B**): Measurement of RA, LA, RV and LV on four-chamber views. (**C**): Measurement of the septal angle. (**D**): Measurement of intraventricular septal (IVS) angle. (**E**): Measurement of IVC. (**F**): The green area is EFV.

Imaging variables were derived from routine preoperative chest CT scans performed upon admission following the onset of AIS. The specific cardiac parameters analyzed in this study were selected based on their established correlation with hemodynamic changes and cardiac function [21,25,26,27,28,29]. Furthermore, these parameters are readily identifiable and reproducible on non-ECG-gated chest CT scans, which aligns perfectly with the imaging protocol utilized in this retrospective study [22]. CT scans were optimized for the mediastinal window (window width: 300 HU, window level: 35 HU) to facilitate the measurement of cardiovascular-related parameters (refer to Figure 2). The maximum diameters of the right and left ventricles/atria indicated the utmost vertical distance between the endocardium and the septum/atrial septum at the level of maximum diameter. As routine preoperative chest CT cannot distinguish between the ventricular wall and the heart chambers, the maximum diameter of the left ventricle encompassed both the left ventricular lateral wall and the septum, while the maximum diameter of the right ventricle included the right ventricular lateral wall. The diameters of the main pulmonary aorta (MPA) and the right/left pulmonary arteries (LPA/RPA) signified the maximum wall-to-wall distances, and the ascending aorta (AA) diameter was measured on the same slice as the MPA [25]. The septal angle represented the angle between the ventricular septum and the midline of the chest, defined as the line connecting the midpoint of the sternum and the spinous process of the thoracic vertebrae [26]. The intraventricular septal (IVS) angle was the angle formed between the point of ventricular insertion and the midpoint of the ventricular septum [27]. These parameters were independently measured in the Picture Archiving and Communication System by two experienced radiologists, who maintained confidentiality of clinical data. In cases of disagreement, a third adjudicator was consulted for a final decision, which was resolved through majority consensus. Epicardial fat volume (EFV) was manually outlined by MZ, who was blinded to both imaging and clinical data, using 3D slicer software version 4.10.2. Yasunori Nagayama et al. demonstrated that NCCT could reliably quantify EFV compared to coronary CT angiography [28]. Other variables, including a history of pneumonectomy, calcification of the aortic arch, coronary artery, mitral valve, and aortic valve, were directly observed.

### 2.4. Statistical Analyses

Data were analyzed using the IBM SPSS statistics software (V. 31.0, IBM, Chicago, IL, USA), with a significance threshold of *p* < 0.05 (two-tailed). Normally and abnormally distributed continuous variables were analyzed using Student’s *t*-test and Mann–Whitney U test, respectively, and were represented as mean ± standard deviation and median (interquartile range). Moreover, categorical variables were analyzed using the χ^2^ test or Fisher’s exact test, which were represented as numbers or percentages. Multivariate analysis employed binary logistic regression to evaluate independent predictors of USR. Variables with bilateral *p*-values < 0.1 from univariate analysis were selected for logistic regression analysis using a backward stepwise approach; we have standardized the RV/A ratio (converted to Z-scores) in the logistic regression model. Model assessment was based on the resulting model, calculating the 95% confidence intervals (CIs) and the area under the curve (AUC). Forest plots were generated to depict independent factors influencing recanalization, alongside nomograms for individualized USR risk assessment. The nomogram’s discrimination and calibration were assessed using calibration curves and decision curve analysis.

## 3. Result

The study encompassed 215 patients with a median age of 72 years, of whom 63.7% were male. A successful recanalization rate of 80% (*n* = 172/215) was achieved. An overview of baseline characteristics is presented in Table 1. Hypertension was prevalent in 65.1% (*n* = 140/215) of the total cohort, with 25.1% (*n* = 54/214) having a history of heart failure, 9.3% (*n* = 20/215) experiencing coronary artery disease, and a median admission NIHSS score of 15 (IQR: 10, 20) and Admission GCS score of 10 (IQR: 7, 13).

Turning to cardiac parameters obtained from routine preoperative chest CT, Table 1 revealed that the mean septal angle was 44.05 ± 10.87, the median intraventricular septal angle was 150.51 (IQR: 142.55, 161.92), and the median epicardial fat volume was 81.6 cubic centimeters (IQR: 52.77, 112.58). Intriguingly, 85.6% (*n* = 184/204) of the final cohort exhibited abnormal electrocardiogram findings, although not all ECG abnormalities necessitate clinical intervention.

Among the 215 patients, unsuccessful recanalization after thrombectomy were classified into the USR group, while the others were classified into the SR group. From Table 2, we clearly revealed that, in the USR group, patients tended to have higher mean arterial pressure (105.86 ± 24.1 vs. 96.85 ± 17.73; *p* = 0.006), larger main pulmonary artery diameter (30.83, IQR: 28, 32.94 vs. 30.46, IQR: 28.23, 33.17; *p* = 0.05), higher RV/A (0.82 ± 0.15 vs. 0.77 ± 0.15; *p* = 0.028), and increased IVS angle (147.75, IQR: 137.79, 160.75 vs. 145.02, IQR: 139.22, 159.01; *p* = 0.019) than the SR group. Conversely, the USR group exhibited a smaller left pulmonary artery diameter (20.43, IQR: 18.51, 22.03 vs. 21.3, IQR: 19.76, 23.52; *p* = 0.008) and MPA/AA (0.78 ± 0.14 vs. 0.84 ± 0.13; *p* = 0.009) than the SR group. However, there was no evidence to suggest that epicardial fat volume (cm^3^) had statistical significance between the two groups (0.78 ± 0.14 vs. 0.84 ± 0.13; *p* = 0.555). Additionally, the SR group consisted of 64.5% male, median age was 68, while the USR consisted of 60.5% male, median age was 72. Although there was a greater proportion of female patients in USR group and they were older, these differences were not statistically significant.

**Figure 3:**
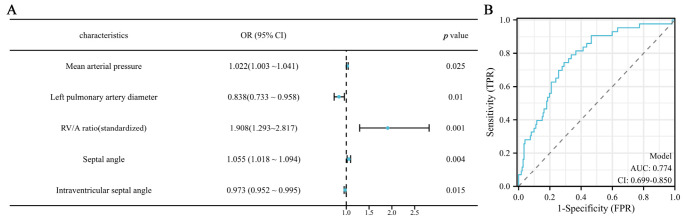
(**A**): Forest plot of the predictors of unsuccessful recanalization. (**B**): Receiver operating characteristic curve of the model. The 95% confidence intervals for the AUC values are represented in parentheses. AUC, area under the curve.

In the multivariate analysis, variables with *p*-values < 0.1 were incorporated into the regression model. The final regression model revealed that the mean arterial pressure (OR: 1.022, CI: 1.003–1.041, *p* = 0.025), left pulmonary artery diameter (OR: 0.838, CI: 0.733–0.958, *p* = 0.010), RV/A ratio (standardized) (OR: 1.908, CI: 1.293–2.817, *p* = 0.001), septal angle (OR: 1.055, CI: 1.018–1.094, *p* = 0.004), and IVS angle (OR: 0.973, CI: 0.952–0.995, *p* = 0.015) were identified as independent risk factors. Furthermore, the receiver operating characteristic curve analysis yielded an area under the curve of 0.774 (95% CI: 0.699–0.850, *p* < 0.001) (Figure 3), suggesting moderate predictive performance of the model.

**Figure 4:**
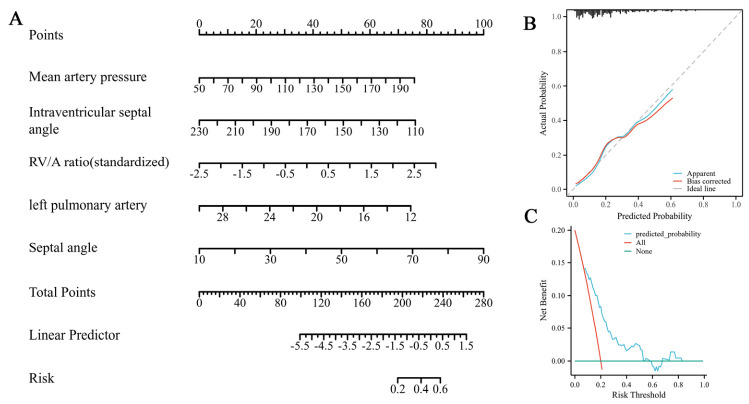
(**A**): The radiological nomogram and its predictive performance for unsuccessful recanalization in patients with acute ischemic stroke. (**B**): Calibration curve of the radiological nomogram, which shows that the probability of unsuccessful recanalization predicted by the nomogram was highly consistent with the actual probability. (**C**): Decision curve analysis for the radiological nomogram, the blue line and the red line represented the assumption that all patients have unsuccessful recanalization and successful recanalization. The green line meant the net benefit of the models.

To predict recanalization following MT for acute ischemic stroke, we developed a nomogram that integrates independent risk factors, including the left pulmonary artery diameter et al. (Figure 4A). The calibration curve showed that the probability of USR predicted by the nomogram was highly consistent with the actual probability (Figure 4B), and decision curve analysis demonstrated favorable net benefits over a range of probability thresholds (Figure 4C), indicating the nomogram’s reliability as a predictive tool.

## 4. Discussion

Numerous randomized controlled trials have substantiated the safety and efficacy of endovascular therapy within 24 h of stroke onset in patients with large vessel occlusion acute ischemic stroke (AIS) [3,4,5,6]. Nevertheless, not all embolization procedures result in successful reperfusion [8,9]. Earlier studies have explored multiple factors influencing recanalization [10,11,12,13]. Yet, several of these parameters demand specialized testing that is often impractical in the acute setting [14]. Recent studies have begun to unravel how cardiac and brain disease influence each other [15,16,17,18,19,20]. To address this challenge, we were seeking some more efficient and convenient ways to obtain cardiovascular parameters for assessing recanalization in AIS patients undergoing MT. These cardiovascular parameters are derived from routine preoperative chest CT images obtained during routine examinations upon patient admission, and they do not incur additional costs, time or expose patients to more radiation from imaging examinations. The acquisition of these indicators is simple, direct, and convenient. We employed these cardiac parameters to develop a nomogram. The nomogram serves as a valuable tool, allowing neuro-interventional clinicians to anticipate and predict the likelihood of successful recanalization prior to embolization procedures. Characterized by its rapid and accessible nature, this method yielded an area under the curve (AUC) of 0.774, indicating its potential utility as an efficient clinical tool.

In our study, a logistic regression model revealed that mean arterial pressure, IVS angle, left pulmonary artery diameter, RV/A, and septal angle, as cardiac radiological indicators, were independent risk factors for unsuccessful recanalization (USR) after thrombectomy in AIS patients. Notably, higher mean arterial pressure often indicates a history of hypertension [29]. It has been demonstrated that hypertension can predict unsuccessful recanalization in patients with cerebral infarction [30], potentially linked to the prevalence of elongated and tortuous vessels [31]. Furthermore, hypertension can lead to atherosclerosis and poor collateral circulation, reducing the likelihood of successful revascularization through embolization [32]. Additionally, elevated mean arterial pressure places a greater burden on the heart and blood vessels, potentially resulting in compensatory cardiac hypertrophy, cardiac insufficiency, and even heart failure. Heart failure, which exacerbates stroke severity, can significantly impact USR [33]. The latest guidelines suggest keeping blood pressure below 180/105 mmHg in AIS patients who are eligible for emergency reperfusion therapy and in the post-thrombolytic phase [3]. Moreover, greater systolic blood pressure variability was associated with an increased risk of stroke recurrence [34], neurologic deterioration [35], and poor long-term functional outcome [36]. Our study suggests that for each 1 mmHg increase in mean arterial pressure, the risk of unsuccessful recanalization is 1.022 times higher.

Among the cardiac parameters measured in AIS patients, the increase in septal angle and the elevation of RV/A ratio are independent risk factors for recanalization failure after MT in patients with AIS. An increased septal angle and RV/A ratio were indicative of an enlarged right ventricle and increased pulmonary pressure, which revealed a good capacity to predict severe hemodynamic status [37]. Previous studies have confirmed that the septal angle is superior to RV/A ratio in assessing pulmonary vascular resistance [26]. Several reports have established an association between higher pulmonary artery pressure and an increased risk of paradoxical embolism, atrial fibrillation, and cerebral venous congestion, all of which are independent stroke risk factors [38,39], which may lead the recanalization failure.

There was a negative correlation between the IVS angle and left ventricular filling rate, left ventricular end-diastolic volume [40]; the smaller the IVS angle, the larger the left ventricular end-diastolic volume, indicating an increase in left ventricular end-diastolic transverse diameter. Cardiac-remodeling (REM) was defined when there is a >20% increase in left ventricular end-diastolic volume (LVEDV) [41]. The process of REM is usually accompanied by an increase in left ventricular volume and a reduction in LVEF [42]. Impaired cardiac function can aggravate pre-existing brain lesions while also triggering new cerebrovascular events [20].

Our study found that left pulmonary artery diameter was also an independent risk for USR. Interestingly, the increased diameter of the left pulmonary artery was observed to facilitate the successful recanalization. We have not found any research on the correlation between pulmonary artery diameter and recanalization after MT in AIS patients. Previous study evaluating pulmonary artery diameter has observed that while pulmonary artery enlargement is not necessarily an indicator of increased pulmonary artery pressure, it may be related to an increase in pulmonary circulation volume [43]. Augmented pulmonary circulation boosts venous return to the left heart, translating into higher cerebral blood flow, enhancing cerebral perfusion [44] and thereby increasing the recanalization rate after MT.

While electrocardiography (ECG)-gated cardiac CT is traditionally favored for minimizing motion artifacts to ensure precise assessment of cardiac morphology and function, recent strides in multidetector CT technology have significantly mitigated these artifacts in non-ECG-gated chest CT scans. This technological evolution has enhanced the detectability and reporting of clinically relevant cardiac findings in routine thoracic imaging [45,46]. Moreover, in the setting of acute stroke, preoperative assessment of cardiac function via ECG-gated CTA or echocardiography may not be feasible. Routine preoperative chest CT, a standard pre-admission and preoperative assessment modality for stroke patients, not only facilitates evaluation of pulmonary status but also reveals numerous incidental findings, particularly within the cardiovascular system [47].

To our knowledge, our study is the first to explore the connection between cardiac parameters and recanalization after thrombectomy. Incidental information obtained from routine preoperative chest CT scan can be valuable for health assessment, prevention, and risk analysis in AIS patients [48]. Our study demonstrated that certain cardiac parameters derived from routine preoperative chest CT were valuable tools for estimating recanalization after MT in AIS patients.

‘Time is brain,’ and any clinical tool must be seamlessly integrated into the workflow without introducing delays. Crucially, we argue that the parameters investigated in this study do not represent a time-consuming hurdle, but rather a time-saving asset. The non-contrast CT chest imaging utilized in our analysis consists of routine preoperative chest CT scans obtained as part of the standard surgical workup. Therefore, extracting these morphological predictors requires no additional scanning time, no extra contrast administration, and no delay in the patient’s transfer to the angiography suite.

While our retrospective cohort had a limited sample size, the potential of this approach lies in its ability to transform passive imaging data into actionable intelligence. It provides a simple and rapid means to extract clinically relevant cardiovascular information for patients. Given the clearly defined data acquisition process, this study can be readily replicated at other centers with minimal difficulty. In the acute setting, possessing such a rapid and straightforward predictive model can significantly optimize clinical workflows and ultimately improve patient outcomes.

### Limitation

Our study has several limitations. Firstly, being retrospective in nature, it may contain omissions or judgment errors in recanalization determination. Additionally, the study’s relatively small sample size and single-center design might limit its generalizability. Conducting multicenter studies with larger sample sizes in the future may effectively mitigate this bias and enhance the external validity of the results. Although the radiological nomogram performed well in terms of ROC curve, calibration, and decision curve analysis (DCA), it warrants further validation in future studies; meanwhile, this study was limited by the isolated assessment of cardiac parameters without concurrent neuroimaging data, and future research should integrate both cardiovascular and cerebral metrics in larger cohorts to validate their combined value in predicting recanalization outcomes. Third, the study did not explore the clinical outcomes following recanalization, and patients with SR may still have poor prognoses. Last but not least, we did not account for differences in operator experience, thrombectomy techniques and devices used, the number of passes performed, or anatomical variations such as brachiocephalic vessel tortuosity. This lack of standardization may introduce performance bias and affect the generalizability of the study results. In future research, incorporating these factors into the analysis would yield more rigorous conclusions.

## 5. Conclusions

This study demonstrates that decreased IVS angle, left pulmonary artery diameter, and increased RV/A, septal angle, and mean arterial pressure adversely affect USR after MT in AIS patients. Nomograms based on these cardiac parameters measured by routine preoperative NCCT exhibit strong predictive capabilities, assisting clinicians in early identification of patients at risk of recanalization failure and facilitating timely, appropriate treatment decisions.

## Figures and Tables

**Table 1:** Baseline characteristics on admission.

Variables	Overall Cohort (*n* = 215)
**Demographics**	
Age, median (IQR)	72 (62, 78)
Gender (male/total)	63.7% (*n* = 137/215)
**Clinical variables**	
Current drinking	35.3% (*n* = 76/215)
Current smoking	35.3% (*n* = 76/215)
Hypertension	65.1% (*n* = 140/215)
Diabetes mellitus	18.6% (*n* = 40/215)
History of cardiac surgery	10.7% (*n* = 23/214)
Previous stroke	8.8% (*n* = 19/214)
Use of antiplatelet drugs	5.1% (*n* = 11/214)
Use of anticoagulation drugs	7.4% (*n* = 16/215)
Lengths-of-stay in hospital, median (IQR)	13 (9, 22)
**Coexisting disease**	
Atrial fibrillation	25.1% (*n* = 54/214)
Coronary artery disease	9.3% (*n* = 20/215)
Pericardial effusion	15.8% (*n* = 34/211)
Pleural effusion	56.3% (*n* = 121/215)
Emphysema	9.3% (*n* = 20/215)
Lung cancer	0.9% (*n* = 2/215)
**Laboratory index**	
White blood cell counts, median (IQR)	8.66 (6.73, 10.78)
Red blood cell counts, median (IQR)	4.51 (4.21, 4.89)
Platelet counts, median (IQR)	203 (166, 252.25)
Random glucose, median (IQR)	7.10 (6.30, 9.15)
Prothrombin time, median (IQR)	13.20 (12.60, 13.90)
Prothrombin activity, median (IQR)	98 (87, 109)
International normalized ratio, median (IQR)	1.01 (0.95, 1.09)
Fibrinogen, median (IQR)	3.24 (2.68, 3.75)
Activated partial thromboplastin time, median (IQR)	34.30 (30.90, 37.50)
APTT ratio, median (IQR)	0.95 (0.85, 1.04)
Thrombin time, median (IQR)	17 (16.20, 18.30)
D-dimer, median (IQR)	0.73 (0.43, 1.62)
Admission systolic blood pressure, median (IQR)	133 (116, 155)
Admission diastolic blood pressure, median (IQR)	78 (68, 90)
Mean arterial pressure, mean ± SD	40.3 ± 6.5
Temperature, median (IQR)	37.40 (37.10, 27.80)
Heart rate, median (IQR)	76.86 (67.21, 92.27)
Abnormal electrocardiogram	85.6% (*n* = 184/204)
Admission NIHSS score, median (IQR)	15 (10, 20)
Admission GCS score, median (IQR)	10 (7, 13)
**Cardiac imaging variables**	
Maximum left ventricular diameter, median (IQR)	65.75 (61.42, 70.95)
Maximum right ventricular diameter, mean ± SD	40.26 ± 6.48
Maximum the left atrium diameter, mean ± SD	46.87 ± 9.66
Maximum the right atrium diameter, mean ± SD	52.69 ± 8.86
Maximum ascending aorta diameter, mean ± SD	36.63 ± 4.27
Aortic arch calcification	80% (*n* = 172/215)
Main pulmonary artery diameter, median (IQR)	30.07 (27.24, 32.68)
Right pulmonary artery diameter, median (IQR)	23.07 (20.99, 25.26)
Left pulmonary artery diameter, median (IQR)	21.25 (19.59, 23.30)
Superior vena cava diameter, mean ± SD	21.8 ± 3.4
Inferior vena cava diameter, median (IQR)	24.29 (22.46, 28.01)
Septal angle, mean ± SD	44.05 ± 10.87
Intraventricular septal angle, median (IQR)	150.51 (142.55, 161.92)
Epicardial fat volume (cm^3^), median (IQR)	81.60 (52.77, 112.58)
Coronary artery calcification	64.2% (*n* = 138/215)
Mitral valve calcification	9.3% (*n* = 20/213)
Aortic valve calcification	8.4% (*n* = 18/214)
History of lung resection surgery	0.5% (*n* = 1/215)

SD, standard deviation; IQR, interquartile range; APTT, activated partial thromboplastin time, median; NIHSS, National Institutes of Health Stroke Scale; GCS, Glasgow coma scale; continuous variables were expressed as mean ± standard deviation or median (IQR). Categorical variables were expressed as counts and percentage.

**Table 2:** Predictors of unsuccessful recanalization: univariable analysis.

Variables	Unsuccessful Recanalization (USR) (*n* = 43/215)	Successful Recanalization (SR) (*n* = 172/215)	*p* Value
**Demographics**			
Age, median (IQR)	71 (58.75, 74.25)	69 (61, 77)	0.335
Gender (male/total)	60.5% (*n* = 26/43)	64.5% (*n* = 111/172)	0.62
**Clinical variables**			
Current drinking	37.2% (*n* = 16/43)	34.9% (*n* = 60/172)	0.775
Current smoking	30.2% (*n* = 13/43)	36.6% (*n* = 63/172)	0.433
Hypertension	67.4% (*n* = 29/43)	64.5% (*n* = 111/172)	0.721
Diabetes mellitus	11.6% (*n* = 5/43)	20.3% (*n* = 35/172)	0.189
History of cardiac surgery	14% (*n* = 6/43)	9.9% (*n* = 17/172)	0.628
Previous stroke	4.7% (*n* = 2/43)	9.9% (*n* = 17/172)	0.429
Use of antiplatelet drugs	9.3% (*n* = 4/43)	4.1% (*n* = 7/171)	0.319
Use of anticoagulation drugs	7% (*n* = 3/43)	7.6% (*n* = 13/172)	1
Lengths-of-stay in hospital, median (IQR)	16 (10, 25)	15 (10, 28)	0.642
**Coexisting disease**			
Atrial fibrillation	27.9% (*n* = 12/43)	24.6% (*n* = 42/172)	0.652
Coronary artery disease	4.7% (*n* = 2/43)	10.5% (*n* = 18/172)	0.379
Pericardial effusion	9.3% (*n* = 4/43)	19.8% (*n* = 34/172)	0.108
Pleural effusion	46.5% (*n* = 20/43)	58.7% (*n* = 101/172)	0.149
Emphysema	11.6% (*n* = 5/43)	8.7% (*n* = 15/172)	0.769
Lung cancer	0% (*n* = 0/43)	1.2% (*n* = 2/172)	1
**Laboratory index**			
White blood cell counts, median (IQR)	8.68 (8.14, 10.46)	8.21 (6.33, 10.33)	0.219
Red blood cell counts, median (IQR)	4.38 (4.22, 4.84)	4.53 (4.21, 4.91)	0.75
Platelet counts, median (IQR)	227.5 (181, 299)	204 (164, 251)	0.156
Random glucose, median (IQR)	6.7 (6.05, 8.3)	7 (6, 9.2)	0.158
Prothrombin time, median (IQR)	13.3 (12.75, 13.93)	13.1 (12.3, 13.8)	0.502
Prothrombin activity, median (IQR)	95.5 (86.5, 105.25)	100 (90, 113)	0.54
International normalized ratio, median (IQR)	1.03 (0.97, 1.1)	1 (0.93, 1.07)	0.519
Fibrinogen, median (IQR)	3.12 (2.45, 3.51)	3.21 (2.73, 3.78)	0.916
Activated partial thromboplastin time, median (IQR)	33.2 (30.85, 36.28)	33.9 (30.2, 36)	0.212
APTT ratio, median (IQR)	0.92 (0.86, 1.01)	0.93 (0.84, 1)	0.188
Thrombin time, median (IQR)	17.05 (16.13, 17.8)	16.7 (15.7, 17.7)	0.321
Thrombin time ratio, median (IQR)	1.01 (0.95, 1.04)	0.98 (0.92, 1.04)	0.345
D-dimer, median (IQR)	0.61 (0.45, 1.75)	0.71 (0.32, 1.08)	0.388
Mean arterial pressure, mean ± SD	105.86 ± 24.1	96.85 ± 17.73	0.006 *
Temperature, median (IQR)	37.6 (37.15, 38)	37.4 (37.2, 37.7)	0.417
Heart rate, median (IQR)	84.64 (70.1, 121.54)	75.21 (66.45, 85.31)	0.217
Abnormal electrocardiogram	95% (*n* = 38/40)	89% (*n* = 146/164)	0.399
Admission NIHSS score, median (IQR)	23 (14.25, 26.25)	13 (8, 19)	0.186
Admission GCS score, median (IQR)	7 (6.75, 11.25)	10 (7, 12)	0.312
**Chest imaging variables**			
Maximum left ventricular diameter, median (IQR)	64.89 (63.66, 67.98)	67.97 (62.73, 75.53)	0.392
Maximum right ventricular diameter, mean ± SD	41.19 ± 7.18	40.08 ± 6.31	0.292
Maximum diameter of the left atrium, mean ± SD	45.76 ± 8.83	47.16 ± 9.86	0.395
Maximum diameter of the right atrium, mean ± SD	51 ± 10	53.12 ± 8.57	0.163
Maximum diameter of ascending aorta, mean ± SD	37.34 ± 4.23	36.48 ± 4.28	0.222
Calcification of the aortic arch	79.1% (*n* = 34/43)	80.2% (*n* = 138/172)	0.865
Main pulmonary artery diameter, median (IQR)	30.83 (28, 32.94)	30.46 (28.23, 33.17)	0.050 *
Right pulmonary artery diameter, median (IQR)	22.59 (19.98, 25.9)	23.01 (20.64, 24.51)	0.082
Left pulmonary artery diameter, median (IQR)	20.43 (18.51, 22.03)	21.3 (19.76, 23.52)	0.008 *
Superior vena cava diameter, mean ± SD	21.03 ± 2.81	21.98 ± 3.56	0.106
Inferior vena cava diameter, median (IQR)	24.49 (22.74, 28.1)	24.24 (22.34, 27.96)	0.774
RV/A, mean ± SD	0.82 ± 0.15	0.77 ± 0.15	0.028 *
LV/A, median (IQR)	1.51 (1.29, 1.69)	1.51 (1.33, 1.76)	0.947
RV/LV, mean ± SD	0.63 ± 0.12	0.60 ± 0.10	0.173
RA/LA, median (IQR)	1.1 (0.99, 1.22)	1.17 (1.02, 1.3)	0.383
MPA/AA, mean ± SD	0.78 ± 0.14	0.84 ± 0.13	0.009 *
Septal angle, mean ± SD	46.56 ± 11.67	43.43 ± 10.63	0.09
Intraventricular septal angle, median (IQR)	147.75 (137.79, 160.75)	145.02 (139.22, 159.01)	0.019 *
Epicardial fat volume (cm^3^), median (IQR)	66.57 (40.98, 128.58)	67 (41.16, 100.8)	0.555
Coronary artery calcification	62.8% (*n* = 27/43)	64.5% (*n* = 111/172)	0.831
Mitral valve calcification	4.8% (*n* = 2/43)	10.5% (*n* = 18/172)	0.394
Aortic valve calcification	7.1% (*n* = 3/43)	8.7% (*n* = 15/172)	0.984
History of lung resection surgery	0% (*n* = 0/43)	0.6% (*n* = 1/172)	1

Comparisons that are statistically significant (*p*  ≤  0.05) are marked with one asterisk. SD, standard deviation; IQR, interquartile range; APTT, activated partial thromboplastin time, median; NIHSS, National Institutes of Health Stroke Scale; GCS, Glasgow coma scale; RV/A, ratio of right ventricle to right atrium; LV/A, ratio of left ventricle to left atrium; RV/LV, ratio of right ventricle to left ventricle; RA/LA, ratio of right atrium to left atrium; MPA/AA, ratio of main pulmonary artery to ascending aorta. Continuous variables were expressed as mean ± standard deviation or median (IQR). Categorical variables were expressed as counts and percentage.

## Data Availability

The data presented in this study are available on request from the corresponding authors.
